# Mr. Ring, in Reply

**Published:** 1804-09-01

**Authors:** John Ring

**Affiliations:** New Street, Hanover-Square


### *To the Editors of the Medical and Physical Journal.*

GENTLEMEN,

THE article in your last Journal, in which my answer to Mr. Goldson is reviewed, requires several observations, which would be too long for you to insert; but I beg the favour of you to insert the following:

A temporary insusceptibility of the small-pox is so common, that I am still inclined to think it rash, and even ridiculous, to conclude, that because a person, at *one period* of life, resists the infection, he will *always* resist it.

I never stated, that I had inoculated eleven hundred of my cow-pock patients with variolous matter; and the Reviewer is also incorrect in asserting, that I had only adduced two instances of danger from the practice. The case of Mr. Miles, and that of Mr. Grant's child, are instances of the contrary; and Dr. Buchan says, he has known several facts of the kind, but does not mention the particulars.

Other instances of the same sort have occurred, some of them them very lately; which I shall shortly lay before the public, in a second edition of my Pamphlet.

I have never discouraged experiments for ascertaining the efficacy of vaccination; but the experiments I prefer are different from those which the Reviewer recommends. I consider variolous inoculation after the cow-pock as no longer necessary; but recommend an experiment, which is more safe, and no less convincing, that of exposure to the natural infection of the small-pox. I am, &c.

*New Street, Hanover-Square,*

*August* 15, 1804.

JOHN RING.

